# Supplementary material for: Sappanone A Prevents Left Ventricular Dysfunction in a Rat Myocardial Ischemia Reperfusion Injury Model
Source: Int J Mol Sci. 2020 Sep 21;21(18):6935. doi: 10.3390/ijms21186935 (PMC7555706; doi:10.3390/ijms21186935)
Supplement: Supplementary file 1 [file ijms-21-06935-s001.zip › Supplementary Material.pdf]

Supplementary figure

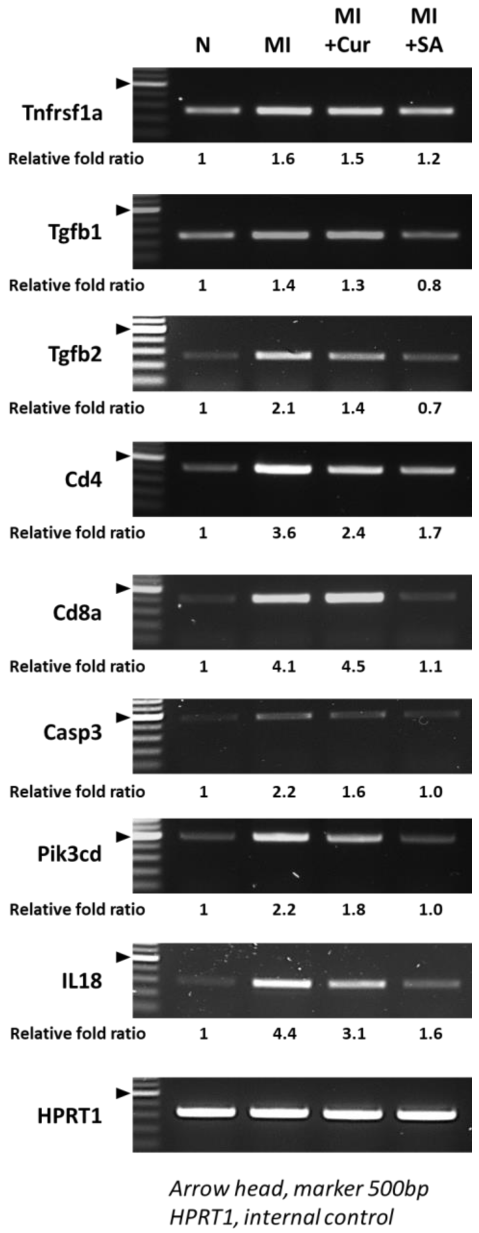

Supplementary Figure S1. Validation of the DEGs involved in the inflammatory responses by RT-PCR.

Supplementary Tables

Supplementary Table S3. mRNA seq results. UMR represents the number of reads uniquely mapped. '% mapped reads' means the percentage of the mapped reads (UMR and multi-mapped reads) over the total sequenced reads. The numbers and percentages of the mapped reads to the mitochondrial genome were also shown.

Sample	Total reads	UMR	Multi-mapped reads	% mapped reads	mapped reads to the MT genome (%)
Normal_1	38,838,450	34,555,742	979,434	91.5	1,626,787 (4.2%)
Normal_2	36,005,890	31,939,265	937,963	91.3	1,508,812 (4.2%)
MI_1	45,246,188	40,746,184	1,688,407	93.8	1,242,781 (2.7%)
MI_2	34,917,396	31,272,122	931,641	92.2	1,403,589 (4.0%)
MI+Cur_1	36,082,672	31,988,882	1,062,074	91.6	1,383,980 (3.8%)
MI+Cur_2	42,613,114	37,954,412	1,424,065	92.4	1,394,025 (3.3%)
MI+SA_1	43,818,952	38,757,713	1,222,794	91.2	1,516,899 (3.5%)
MI+SA_2	44,381,526	39,266,038	1,231,531	91.2	1,555,887 (3.5%)

Supplementary Table S4. List of the primers used in this study.

Symbol	Primer direction	Primer sequence [5'->3']	Primer Tm(℃)	Annealing Tm(℃)	PCR cycles	Product size(bp)
Tnfrsf1a	forward	AAGTGCCACAAAGGAACCTAC	58.42	58	22	255
	reverse	GTCCACACACTGGAAATGCG	59.76			
Tgfb1	forward	TGACATGAACCGACCCTTCC	59.68	58	22	245
	reverse	CCAGGCTCCAAATGTAGGGG	60.11			
Tgfb2	forward	CTCAGTGGGCAGCTTTTGCT	61.18	59	27	262
	reverse	AATGTAGCGCTGGGTTGGAG	60.39			
Cd4	forward	AAGGACTGGCCAGAGACTCA	60.18	58	27	397
	reverse	TTCTTGTTCTCCAGCTCGCA	59.61			
Cd8a	forward	CCTTGTCAAGCCCAGACCTT	59.89	59	27	399
	reverse	CAGCTTGGGTTCTCTCCTGG	60.04			
Casp3	forward	GGAGCTTGGAACGCGAAGAA	60.95	59	27	473
	reverse	GGCAGTAGTCGCCTCTGAAG	60.18			
Pik3cd	forward	CATCAAGTCCCTACGGAAGC	58.06	58	27	478
	reverse	TTTGGAGTCCATGAAGGTGC	58.09			
Il18	forward	CTGATATCGACCGAACAGCC	58.23	57	27	296
	reverse	AGCATCATCTTCCTTTTGGCA	58.19			
HPRT1	forward	ATACAGGCCAGACTTTGTTGGA	59.62	58	27	330
	reverse	GCTGCCTACAGGCTCATAGT	59.24			

Tables S1 and S2. See the attached excel file.

Supplementary Table S1. 3568 DEGs from the three comparisons (MI versus normal, MI + Curcumin versus MI, and MI + Sappanone A versus MI). Ensembl IDs, Entrez IDs, symbols, descriptions, P-values, and log2-fold-changes of the DEGs are shown. P-values were computed as described in Materials and Methods section

Supplementary Table S2. Histopathologic individual data. (A) Histopathologic individual data of rat heart tissues by grading. Grades 1, 2, 3, and 4 show minimal, slight, moderate, and severe pathological changes, respectively. Values are mean ± standard deviation (SD, *n*=3). (B) Histopathologic individual data of each cell type of inflammatory cells infiltration. Mixed cell, lymphocyte, mononuclear cell, purulent lesions were described.
